# Yin Yang 1 is associated with cancer stem cell transcription factors (SOX2, OCT4, BMI1) and clinical implication

**DOI:** 10.1186/s13046-016-0359-2

**Published:** 2016-05-25

**Authors:** Samantha Kaufhold, Hermes Garbán, Benjamin Bonavida

**Affiliations:** Department of Microbiology, Immunology, and Molecular Genetics, David Geffen School of Medicine, University of California Los Angeles, Los Angeles, CA 90095 USA; NantBioScience, Inc, NantWorks, LLC & California NanoSystems Institute (CnSI), University of California Los Angeles, Los Angeles, CA 90095 USA

**Keywords:** BMI1, Cancer stem cells, NANOG, OCT4, SOX2, Yin Yang 1

## Abstract

The transcription factor Yin Yang 1 (YY1) is frequently overexpressed in cancerous tissues compared to normal tissues and has regulatory roles in cell proliferation, cell viability, epithelial-mesenchymal transition, metastasis and drug/immune resistance. YY1 shares many properties with cancer stem cells (CSCs) that drive tumorigenesis, metastasis and drug resistance and are regulated by overexpression of certain transcription factors, including SOX2, OCT4 (POU5F1), BMI1 and NANOG. Based on these similarities, it was expected that YY1 expression would be associated with SOX2, OCT4, BMI1, and NANOG’s expressions and activities. Data mining from the proteomic tissue-based datasets from the Human Protein Atlas were used for protein expression patterns of YY1 and the four CSC markers in 17 types of cancer, including both solid and hematological malignancies. A close association was revealed between the frequency of expressions of YY1 and SOX2 as well as SOX2 and OCT4 in all cancers analyzed. Two types of dynamics were identified based on the nature of their association, namely, inverse or direct, between YY1 and SOX2. These two dynamics define distinctive patterns of BMI1 and OCT4 expressions. The relationship between YY1 and SOX2 expressions as well as the expressions of BMI1 and OCT4 resulted in the classification of four groups of cancers with distinct molecular signatures: **1)** Prostate, lung, cervical, endometrial, ovarian and glioma cancers (YY1^lo^SOX2^hi^BMI1^hi^OCT4^hi^) **2)** Skin, testis and breast cancers (YY1^hi^SOX2^lo^BMI1^hi^OCT4^hi^) **3)** Liver, stomach, renal, pancreatic and urothelial cancers (YY1^lo^SOX2^lo^BMI1^hi^OCT4^hi^) and **4)** Colorectal cancer, lymphoma and melanoma (YY1^hi^SOX2^hi^BMI1^lo^OCT4^hi^). A regulatory loop is proposed consisting of the cross-talk between the NF-kB/PI3K/AKT pathways and the downstream inter-regulation of target gene products YY1, OCT4, SOX2 and BMI1.

**Electronic supplementary material:**

The online version of this article (doi:10.1186/s13046-016-0359-2) contains supplementary material, which is available to authorized users.

## Background

### General properties of cancer stem cells (CSCs)

Drug resistance and metastatic spread are two key characteristics of tumors that make cancer so difficult to eradicate. Tumors are comprised of heterogeneous cell subpopulations, and these subsets respond distinctly and differently to various therapeutics [1]. One subset of these cells consists of cancer stem cells (CSCs), which are largely similar to normal stem cells with respect to both their behavior and their regulation [2]. CSCs are pluripotent, capable of self-renewal, highly resistant to cytotoxic therapies, and drive tumorigenesis.

In addition to different cellular responses, adaptive changes like the epithelial to mesenchymal transition (EMT) exacerbate metastatic spread and drug resistance. EMT is the process by which epithelial cells lose their apico-basolateral polarity to become migratory mesenchymal cells. This process is crucial to embryonic differentiation but is dysregulated in cancer, affording tumor cells invasive and migratory properties. EMT has been shown to cause reversion to a CSC-like phenotype, linking CSCs, EMT and drug resistance [3, 4].

Clearly, better molecular and biochemical understandings of the phenotypic and functional properties of CSCs will help in the development of novel and specific targeted therapeutics to eradicate CSCs. Thus, these should reduce the inherent resistance and relapses and should prolong survival. Below, we briefly present reported studies on the various regulatory factors in the development of CSCs.

### CSC markers

The sex determining region Y-box 2 *(SOX2),* POU class 5 homeobox 1 *(POU5F1*), B cell-specific Moloney murine leukemia virus insertion site 1 *(BMI1*) and Nanog homeobox (*NANOG*) genes are four genes encoding transcription factors that have been reported to be involved in the regulation of CSCs. SOX2, Octamer-binding transcription factor 4 (OCT4) (the transcription factor encoded by *POU5F1*, also known as POU5F1) and NANOG make up the core transcriptional network responsible for the regulation of stem cell self-renewal and pluripotency [5].

#### SOX2

- General properties of SOX2
  The *SOX2* gene is located on chromosome 3q26.3-q27 [6, 7]. The SOX2 protein is composed of 317 amino acids and has a mass of 34.3 kDa [8]. Originally characterized in 1994, SOX2 is a member of the SOXB1 family of transcription factors, and its three primary domains are an N-terminal domain, a high-mobility group (HMG) domain, and a transactivation domain [9]. Protein partners, nuclear import signals, and nuclear export signals bind the HMG domain, while the C-terminal transactivation domain is responsible for promoter binding, causing the activation or repression of target genes [10].
- SOX2 expression in various cancers
  SOX2 is expressed in neural stem cells [11], breast stem cells [12], and stem populations in the liver, pancreas, and stomach [13]. SOX2 overexpression in recurrent prostate cancer tissues has been reported [14]. SOX2 is likewise overexpressed in head and neck squamous cell carcinoma [15]. Bioinformatics analysis showed *SOX2* overexpression in 7/36 solid tumors analyzed [16].
  Multiplication of the 3q26.3 gene locus causes SOX2 amplification, which has been reported in glioblastoma, small-cell lung cancer and many squamous cell carcinomas [17–24]. Co-amplification of SOX2 and Protein Kinase C_I_ (PRKC_I_) has been reported to be responsible for the CSC phenotype in lung squamous cell carcinoma [25]. Additionally, FGF induces SOX2 in osteoblasts [26].
- SOX2 functions
  In pancreatic cancer cells, SOX2 overexpression causes increased cell proliferation via cyclin D3 induction [27]. Subsequent SOX2 knockdown causes transcriptional induction of p21^Cip1^ and p27^Kip1^, resulting in cell cycle arrest and cell growth inhibition [27]. Similarly, SOX2 silencing inhibits cellular proliferation in lung squamous cell carcinoma cells [28]. The upregulation of BMP4, which acts as a tumor suppressor, is responsible for this inhibition of proliferation [28]. SOX2 silencing causes a decrease in cell proliferation and loss of tumorigenicity in glioblastoma tumor-initiating cells in immunodeficient mice [29]. SOX2 has also been reported to promote cellular proliferation in breast, prostate, and cervical cancers, among others [30–32]. Furthermore, SOX2 has been implicated in the evasion of apoptotic signals in prostate cancer, gastric cancer and NSCLC [32–34]. SOX2 has been reported to promote invasion, migration, and metastasis in melanoma, colorectal cancer, glioma, gastric cancer, ovarian cancer and hepatocellular carcinoma [20, 35–38]. SOX2 mediates invasive and migratory phenotypes, in part, through MMP3, MMP2, and PI3K/AKT/mTOR activations [35, 37, 39].
- Regulation of SOX2
  The ubiquitin-specific protease 22 (USP22) represses the SOX2 promoter in embryonic differentiation [40]. Activation of EGFR signaling increases SOX2 expression and self-renewal in prostate CSCs [41]. Furthermore, an EGFR/STAT3/SOX2 signaling pathway has been reported in murine breast cancer stem cells [42]. In primary melanoma cells, GLI1 and GLI2 have been reported to bind the proximal *SOX2* promoter, indicating that SOX2 is regulated, in part, by Hedghog-GLI signaling [43]. The PI3K/Akt signaling pathway has been shown to be activated in prostate cancer cells overexpressing SOX2 [44]. By contrast, ovarian adenocarcinoma cells overexpressing SOX2 have been reported to possess an inhibited PI3K/Akt signaling pathway [45]. These conflicting results suggest that PI3K/Akt modulation may have an important role in the expression of SOX2.
- Clinical implications
  SOX2 overexpression is correlated with tumor recurrence, poor prognosis and chemoresistance in head and neck squamous cell carcinoma [15]. SOX2 overexpression increases tumorigenicity and inhibits differentiation in neuroblastoma [46]. High SOX2 expression is associated with higher histological grade in esophageal squamous cancer (*p* < 0.001) [47]. A significant correlation (*p* < 0.001) between high SOX2 expression and decreasing patient survival was also established in the same study [47]. In contrast, SOX2 has been shown to correlate with improved survival and better patient outcome in lung cancer [48–53]. The expressions of SOX2, OCT4 and NANOG correlate positively with the pathological grade of gliomas [54].

#### OCT4

- General properties of OCT4
  The *POU5F1* gene is located on chromosome 6p21.33 [6, 55]. There are 6 related pseudogenes on chromosomes 1, 3, 8, 10 and 12 [56], and alternative splicing results in multiple protein isoforms. The canonical OCT4 protein sequence contains 360 amino acids and weighs 38.6 kDa [8]. OCT4, also referred to as POUF51 and OCT3/4, is another transcription factor necessary for maintaining pluripotency [57]. The POU domain is the main active domain for OCT4. The POU domain is composed of two subdomains, an amino-terminal POU specific region and a carboxyl-terminal homeodomain, and both bind DNA through helix-turn-helix structures [58].
- OCT4 expression in various cancers
  OCT4 is overexpressed in recurrent prostate cancer tissues [14]. Bioinformatics analysis showed OCT4 overexpression in 9/36 of different solid tumor types when compared to corresponding normal tissues [16]. Namely, OCT4 was overexpressed in bladder, brain, lung, ovarian, pancreatic, prostate, renal, seminoma and testicular cancers. OCT4 overexpression was observed in 1/4 hematological cancers—namely, chronic lymphocytic leukemia—in the same study [16].
- OCT4 functions
  OCT4 works synergistically with SOX2, among other factors, to regulate transcription. SOX2 and OCT4 are both activators of genes involved in pluripotency, including themselves and *NANOG*, and repressors of genes involved in differentiation (e.g., *HOXB1, PAX6, MYF5*) [59, 60]. SOX2 and OCT4 interact directly to activate target gene transcription [61]. OCT4/SOX2 heterodimers bind the *NANOG* proximal promoter region to induce transcription [62, 63].
- Regulation of OCT4
  SOX2 and OCT4 regulate their own transcription by binding the composite sox-oct elements in the *SOX2* and *POU5F1* enhancers [64]. In pluripotent stem cells, Foxm1 directly binds the *POU5F1* promoter −3 kb upstream region [65]. Among others, SALL4, ESRRB, and PAF1 have been implicated as positive regulators of OCT4 expression, while TCF3, GCNF, HIF and CDK2 have been identified as negative regulators [66].
- Clinical implications
  Like high SOX2 expression, high OCT4 expression is associated with higher histological grade in esophageal squamous cancer (*p* < 0.001) [47]. Both NANOG and OCT4 overexpressions are associated with both advanced cancer stage and decreased survival in oral squamous cell and lung adenocarcinomas [67].

#### NANOG

- General properties of NANOG
  The *NANOG* gene is located on chromosome 12p13.31 [6, 7, 55]. The 2184-nucleotide *NANOG* cDNA encodes the NANOG protein [68]. While there are 11 NANOG pseudogenes [69], only pseudogene 8 has an open reading frame capable of producing the functional NANOG protein [69–72]. The canonical NANOG protein has a sequence of 305 amino acids and a mass of 34.6 kDa [8]. Another isoform, NANOG-delta 48, lacks amino acids 168–183 and is, consequently, 289 amino acids long [8].
- NANOG expression in various cancers
  Both pluripotent mouse and human stem cell lines express *NANOG* mRNA, but *NANOG* mRNA is absent from differentiated cells [73]. NANOG protein levels have been reported to be elevated in oral squamous cell carcinoma tumor tissues as compared to corresponding normal tissues (*p* = 0.014) [74].
- NANOG functions
  NANOG is capable of maintaining embryonic stem cell pluripotency independently of the LIF-STAT3 pathway, which OCT4 is incapable of doing [68, 73]. NANOG exerts many functions through its transcriptional regulatory activities. NANOG regulates the cell cycle and proliferation by directly binding the cyclin D1 promoter [75, 76]. In prostate cancer cell lines, the induction of NANOG causes the upregulations of CD133 and ALDH1 [77]. NANOG alone is sufficient to induce SLUG transcription [67]. Furthermore, NANOG is capable of inducing CSC-like properties in primary p53-deficient mature mouse astrocytes; however, astrocytes with intact p53 could not be induced [78]. Some functional redundancy and cooperation between NANOG and STAT3 have been reported. The two form a complex in head and neck squamous cell carcinoma cells [79], and microarray analysis showed that NANOG also regulated 14 out of the 22 STAT3 target genes involved in the maintenance of an undifferentiated state [80].
  SOX2, OCT4 and NANOG co-occupy the promoter regions of at least 353 genes, and NANOG has been shown to occupy >90 % of the promoter regions bound by both OCT4 and SOX2 in human embryonic stem cells [59]. More explicitly, in >90 % of the cases where a promoter region is bound by both OCT4 and SOX2 in human embryonic stem cells, NANOG is also present.
- Regulation of NANOG
  In addition to OCT4/SOX2 heterodimers, NANOG is regulated at a transcriptional level by multiple factors. GLI1 and GLI2 activate *NANOG* transcription by directly binding cis-regulatory sequences of the *NANOG* gene in neural stem cells [81]. In mouse embryonic stem cells, p53 was shown to suppress *NANOG* transcription in response to DNA damage. P53 directly binds the *NANOG* promoter through two consensus binding motifs [82]. LIF-induced STAT3 phosphorylation also results in the upregulation of NANOG in embryonic stem cells [83].
- Clinical implications
  Increased nuclear NANOG expression has been associated with high-grade subtypes of ovarian cancer and poor disease-free survival [84]. Additionally, NANOG overexpression is correlated with poor prognoses for colorectal and breast cancer patients, as well as for ovarian cancer patients [85–87].

#### BMI1

- General properties of BMI1
  Ensembl identifies the *BMI1* gene as located on chromosome 10p12.2. The BMI1 protein is composed of 326 amino acids and has a mass of 36.9 kDa [8]. BMI1 is a member of the polycomb repressive complex 1 (PRC1), which also includes Mel-18, Mph1/Rae28, M33, Scmh1, and Ring 2. The polycomb repressive complex 2 (PRC2) is comprised of EED, EZH, Sux12 and YY1 [88].
- BMI1 expression in various cancers
  BMI1 expression levels have been reported to be high in many tissues, including the brain, esophagus, kidney, lungs, and blood, among others [89]. BMI1 levels are also elevated in various solid tumor-forming cancers, among them neuroblastoma and bladder cancer [90, 91].
- BMI1 functions
  BMI1 is involved in the maintenance and/or self-renewal of many stem cell types, including embryonic, neural, hematopoietic and prostate [92–95]. BMI1 promotes the proliferation of leukemic stem cells in mouse models [96], and BMI1 activates the self-renewal ability of neural stem cells [97].
  BMI1 is directly responsible for the regulation of multiple targets. BMI1 regulates the tumor suppressors p16INK4a and p14ARF [98, 99]. BMI1 also directly binds the *PTEN* promoter, resulting in the activation of the PI3K/AKT pathway and subsequently SNAIL stabilization and EMT induction [100]. Additionally, BMI1 directly occupies the *CDH1* promoter, causing E-cadherin repression [100].
  In endometrial cancer cells, the loss of BMI1 results in the reduced expression of SOX2 and KLF4 [101]. CD133+ breast CSCs with high SLUG expression have been shown to also have high BMI1 expression [102]. Furthermore, BMI1 cooperates with TWIST1 to promote cancer dedifferentiation and metastasis [103]. BMI1 overexpression correlates with NANOG overexpression, high-grade status and increased self-renewal in breast adenocarcinomas [104].
- Regulation of BMI1
  Multiple major regulatory pathways, including Akt, Wnt and Notch, contribute to the regulation of BMI1 [105]. Additionally, the Hedgehog pathway activates BMI1 in breast stem cells [106].
- Clinical implications
  Elevated *BMI1* RNA levels have been correlated with more advanced chronic myeloid leukemia status [96, 107]. Increased levels of BMI1 are correlated with poor prognoses in head and neck cancers [103], including in nasopharyngeal carcinoma patients [108]. BMI1 is further correlated with radio- and chemoresistance in head and neck squamous cell carcinomas and is considered to be a predictive factor for overall survival [109]. In glioma, BMI1 expression inversely correlates with survival and positively correlates with poor prognosis [110]. In non-Hodgkin B-cell lymphoma patients, BMI1 expression is associated with poor outcome as well [111].

## CSC markers’ associations with drug resistance

Each of the CSC markers discussed above are implicated in resistance to cancer treatments. In breast cancer, SOX2 silencing restores tamoxifen sensitivity [112]. NANOG overexpression, likewise, increases drug resistance in breast cancer cell lines [77]. Lung adenocarcinoma cell lines are sensitized to erlotinib by shRNA-knockdown of SOX2 [50]. Overexpressions of NANOG and OCT4 also afford lung adenocarcinoma cells a high tolerance to cisplatin [67]. The overexpression of NANOG promotes cisplatin resistance in esophageal cancer [113], and siRNA-knockdown of NANOG increases sensitivity to cisplatin [114]. The elevated expressions of NANOG and OCT4 correlate with cisplatin resistance and recurrence in oral squamous cell carcinoma [115]. SOX2 has been implicated in paclitaxel resistance in prostate cancer cell lines [44]. Docetaxel sensitivity is increased in prostate cancer cells by silencing BMI1 [116]. Ovarian CSCs are more resistant to cisplatin and paclitaxel when BMI1 is overexpressed [117], and targeting BMI1 sensitizes ovarian cancer cells to cisplatin [118]. Furthermore, CD44+/CD24+ pancreatic cancer cells expressing high levels of BMI1 are largely resistant to gemcitabine [119].

## Yin Yang 1 (YY1) and its relationship with CSC transcription factors

### General characteristics of YY1

Yin Yang 1 (YY1) is an ubiquitously expressed zinc-finger transcription factor encoded by the 23 kb *YY1* gene [120–124]. Comprised of 414 amino acids, YY1 exerts various cellular functions, including transcriptional regulation, cell proliferation, chromatin remodeling and apoptosis [124–128]. YY1 regulates multiple targets, including *ERBB2*, *p53*, caspases and HDACs, which are implicated in cancer progression [127].

YY1 is overexpressed in many types of cancer, including metastatic breast cancer [129, 130], colon cancer [131], gastric cancer [132] and prostate cancer [133]. Patterns of YY1 protein expression levels in human cancers have been reviewed previously [134]. The enrichment of binding sites for YY1 and NANOG was identified in the interactomes of both *SOX2* and *POU5F1* [135]. Additionally, YY1 upregulates NANOG in gastric cancer [136]. YY1 has been previously reported to directly interact with embryonic ectoderm development (EED), a protein of the same PcG family as BMI1 [137].

### YY1 and its relationship to CSC transcription factors

**Fig. 1:**
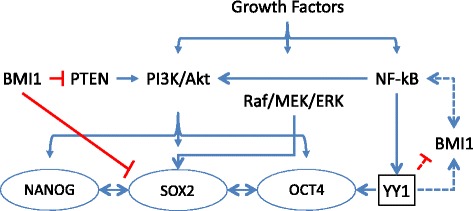
Hypothesized cross-talk between YY1 and CSC transcription factors. This model reflects prior findings and proposed linkages between YY1 and CSC markers

There exist similar patterns of overexpression in various cancerous tissues between the four CSC markers (SOX2, OCT4, BMI1 and NANOG) and YY1. For example, the overexpressions of YY1 [133], SOX2 and OCT4 [14] in prostate cancer cell lines have been reported. These findings suggested that there may be a functional correlation between YY1 and the CSC markers (Fig. 1). Furthermore, the binding site enrichment and interactions with NANOG and EED suggest that YY1 may be associated with the CSC transcription factors [135–137].

### Data mining from proteomic datasets

Publicly available proteomics datasets were used to assess whether YY1, SOX2, OCT4, NANOG and BMI1 expressions correlated in both solid tumors (*n* = 16) and hematological malignancies (*n* = 1) [138].

1. SOX2, OCT4, NANOG and BMI1 were chosen as representative CSC markers because of their well-documented roles in stem cell maintenance as well as data availability [5, 59, 92–95].
  The Human Protein Atlas’ Cancer Atlas feature provides antibody staining information for many proteins in various cancerous tissues [139]. The antibodies used by the Human Protein Atlas for this staining analysis are summarized in Table 1. The Cancer Atlas feature was interrogated to assess the antibody staining of YY1, SOX2, OCT4, NANOG and BMI1 in different cancer types. In each case, the protein of interest was searched in the Cancer Atlas. The resulting “staining overview” presented the antibody staining profile, which differentiated stains into high, medium, low or not detected groups, for the protein in twenty types of cancers, namely, breast, carcinoid, cervical, colorectal, endometrial, glioma, head and neck, liver, lung, lymphoma, melanoma, ovarian, pancreatic, prostate, renal, skin, stomach, testis, thyroid and urothelial cancers. The three cancers with sample sizes fewer than four—carcinoid, head and neck, and thyroid—were discarded, bringing the total types of cancers considered herein to seventeen.

**Table 1:** Selected antibodies characteristics

	YY1 antibody HPA001119	SOX2 antibody CAB010648	NANOG antibody CAB019380	BMI1 antibody CAB011120	OCT4 antibody CAB026380
Provider	Atlas Antibodies, Sigma-Aldrich	Chemicon	SDIX	Upstate	SDIX
Product name	HPA001119	AB5603	2929.00.02	05–637	3542.00.02
Host species	Rabbit	Rabbit	Rabbit	Mouse	Rabbit
Clonality	pAb	pAb	msAb	mAb	msAb
Antigen	Recombinant protein fragment	Synthetic peptide	Genetic immunization	Recombinant protein	Genetic immunization
Cross reactivity	YY2 (80 %)	N/A	N/A	N/A	N/A
2. Example profile
  SOX2 staining qualified as 0/12 high, 1/12 medium, 9/12 low and 2/12 not detected in breast cancer. In this case, 12 different tissues were stained from 12 different patients. A binary system was then applied such that only high or medium scores were considered positive staining. To assess the percentage of positive staining, the number of “positive” (high or medium) stains was divided by the total number of stains performed. In the SOX2 case presented above, (0 high +1 medium)/12 stains yields 1/12 positive staining.
3. Example analysis
  Breast cancer can be used as an example to illustrate the complete process used by us for analysis for one cancer type. The Human Protein Atlas’ Cancer Atlas reported that YY1 antibody HPA001119 yielded 2 high, 5 medium, 1 low and 1 not detected stains in this type of cancer. Consequently, high and medium scores comprised 7 out of a total 9 stains. In this case, 78 % of the stains were above the binary threshold and considered positive staining. YY1 antibody CAB009392 yielded 0 high, 6 medium, 5 low and 0 not detected stains, so the positive staining was 6/11 or 55 %. As previously described, the SOX2 antibody CAB010648 profile was 0 high, 1 medium, 9 low and 2 not detected stains. Only 1/12 stains exceeded the binary threshold for 8 % positive staining. NANOG antibody CAB019380 was never detected in breast cancer and had 0 % positive staining. By these same processes, it was determined that BMI1 antibody HPA030472 had 10/11 (91 %), BMI1 antibody CAB011120 had 8/10 (80 %), OCT4 antibody CAB025600 had 11/11 (100 %) and OCT4 antibody CAB026380 had 5/9 (55 %) positive staining.
4. Antibody selection
  We have selected only one antibody staining profile for each protein that was stained by two different antibodies. This choice was based on the recommendation of the Human Protein Atlas itself and was performed for the sake of simplifying ensuing analyses. The final antibodies used were YY1 antibody HPA001119, SOX2 antibody CAB010648, NANOG antibody CAB019380, BMI1 antibody CAB011120 and OCT4 antibody CAB026380 (Table 1). Of note, YY1 antibody HPA01119 appears to show cross-reactivity with related protein YY2 (based on an 80 % amino acid homology), and thus, cross-reactivity between family members by the various antibodies used must be considered with the results. It is impossible to ignore the possibilities that the noted expression correlations are instead between YY2 and CSC transcription factors, or that there is a functional redundancy between YY1 and YY2 that cannot be distinguished. To that end, the development of YY1-specific antibodies is necessary to validate these findings in vitro. Western blots should be conducted to confirm these findings experimentally.
5. Further analyses
  These procedures were repeated for each antibody in each type of cancer and used to produce binary expression graphs and corresponding statistical analyses. The example breast cancer binary frequency of the protein expression graph was used as a prototype (Fig. 2). The binary expression graphs only show the positive staining derived from the high and medium staining of each antibody in breast cancer. Two-factor without replication analysis of variance was applied to analyze the source of variation between the different antibody staining figures. Pearson correlations were performed to compare the staining of each antibody of each marker to the others. Post-hoc t-tests also assessed the significance between YY1 and CSC marker expressions. From these data, patterns of YY1, SOX2, OCT4, NANOG and BMI1 protein expressions were elucidated and evaluated.

**Fig. 2:**
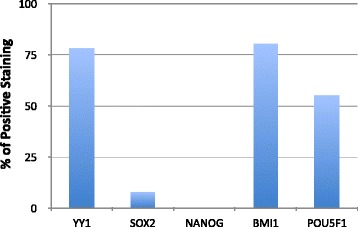
Example of a represented binary percentages of positive staining (frequency of protein expression) in breast cancer. Staining was categorized as high, medium, low or not detected for each antibody by the Human Protein Atlas (proteinatlas.org). For the purposes of applying a binary system of enumeration, only high or medium scores were considered positive staining. The number of these positive stains was divided by the number of total stains performed to ascertain the percentage of positive staining. For example, for YY1 antibody HPA001119, there were 2 high and 5 medium scores. That comprises 7 out of a total 9 stains for a total of 78 % positive staining. The graph compares these percentages of positive staining for each antibody—YY1 antibody HPA001119, YY1 antibody CAB009392, SOX2 antibody CAB010648, NANOG antibody CAB019380, BMI1 antibody HPA030472, BMI1 antibody CAB011120, POU5F1 antibody CAB025600 and POU5F1 antibody CAB026380—in breast cancer

## Clustered expression analyses

Through the grouping of marker expression patterns, our analyses identified four distinct tiers of cancers (Additional file 1: Figure S1). Initially, two distinct dynamics were identified: in clusters one and two, YY1 and SOX2 expressions share an inverse relationship, while in clusters three and four, YY1 and SOX2 expressions have a strong direct correlation. The two types of relationship between YY1 and SOX2 expression appear to dictate distinctive patterns of expression of BMI1 and OCT4. These patterns of expression between YY1, SOX2, OCT4 and BMI1 were used to classify the four tiers.

### Tier 1

**Fig. 3:**
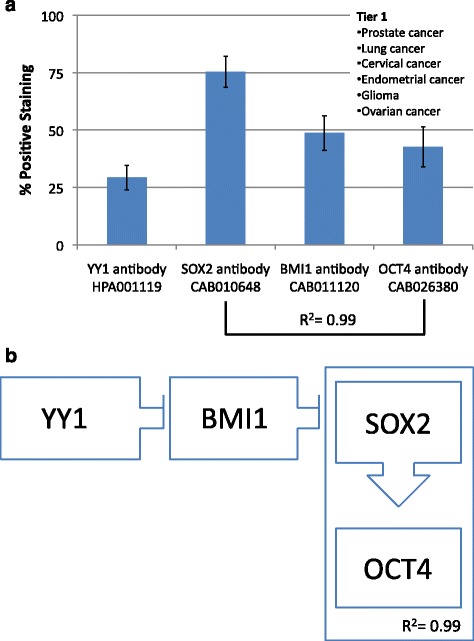
**a** Frequency of Protein expression. Antibody staining for glioma, prostate, lung, cervical, endometrial, and ovarian cancers (Tier 1). SOX2 and OCT4 have a strong direct correlation (R^2^ = 0.99). **b** Hypothetical functional dynamic of CSC-related transcription factors. Correlations of the frequency of protein expression in a segregated malignancies tier

The first group consists of prostate, lung, cervical, endometrial and ovarian cancers as well as glioma. This group shows low YY1 expression with concomitant high SOX2, BMI1 and OCT4 expressions (Fig. 3). Of note, comparison of SOX2 and OCT4 expressions yields an R^2^ value of 0.99, indicating a strong direct correlation.

### Tier 2

**Fig. 4:**
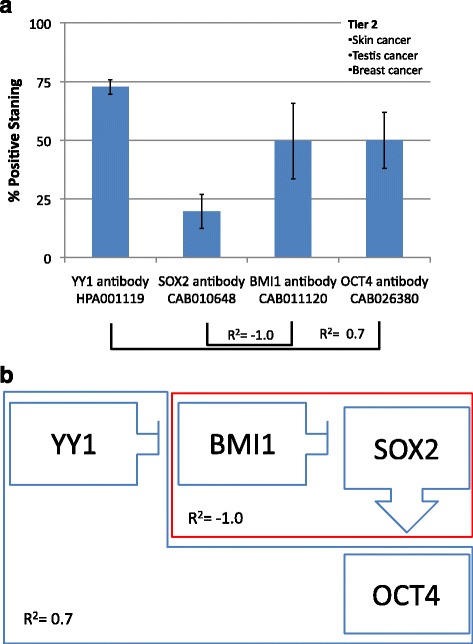
**a** Frequency of Protein expression. Antibody staining for skin, testis and breast cancers (Tier 2). YY1 is positively associated with OCT4 (R^2^ = 0.7), while SOX2 is negatively associated with BMI1 (R^2^ = −1.0). **b** Hypothetical functional dynamic of CSC-related transcription factors. Correlations of the frequency of protein expression in a segregated malignancies tier

Tier two, characterized by high YY1 and low SOX2, is found in skin, testis and breast cancers. SOX2 and BMI1 have a strong inverse correlation (R^2^ = −1.0) and YY1 has a direct correlation with OCT4 expression (R^2^ = 0.7). These findings show an overall pattern of high YY1, low SOX2, high BMI1 and high OCT4 (Fig. 4).

### Tier 3

**Fig. 5:**
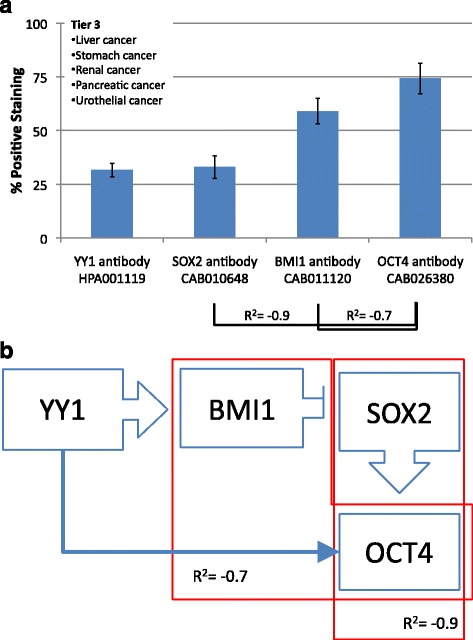
**a** Frequency of Protein expression. Antibody staining for liver, stomach, renal, pancreatic and urothelial cancers (Tier 3). There are significant negative correlations between SOX2 and OCT4 (R^2^ = −0.9) as well as between BMI1 and OCT4 (R^2^ = −0.7). **b** Hypothetical functional dynamic of CSC-related transcription factors. Correlations of the frequency of protein expression in a segregated malignancies tier

Liver, stomach, renal, pancreatic and urothelial cancers make up the third tier. This cluster has low YY1 and low SOX2 expressions. SOX2 and OCT4 have a strong inverse correlation (R^2^ = −0.9), and BMI1 and OCT4 also have an inverse correlation (R^2^ = −0.7). This group has a molecular signature of low YY1 and SOX2 with high BMI1 and OCT4 (Fig. 5).

### Tier 4

**Fig. 6:**
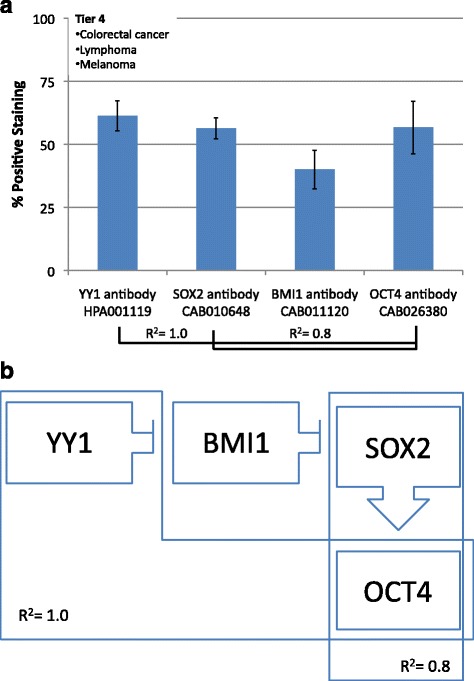
**a** Frequency of Protein expression. Antibody staining for colorectal cancer, lymphoma and melanoma (Tier4). There are strong positive associations between YY1 and OCT4 (R^2^ = 1.0) as well as between SOX2 and OCT4 (R^2^ = 0.8). **b** Hypothetical functional dynamic of CSC-related transcription factors. Correlations of the frequency of protein expression in a segregated malignancies tier

The fourth tier has high YY1 and high SOX2. This group consists of colorectal cancer, lymphoma and melanoma. YY1 and SOX2 both share strong direct correlations with OCT4 expression (R^2^ = 1.0 and 0.8, respectively). In this case, YY1, SOX2 and OCT4 all have high expressions while BMI1 has low expression (Fig. 6).

### Overarching results

Taken together, tiers one and two show a strong inverse correlation between YY1 and SOX2 (R^2^ = −0.9). On the contrary, tiers three and four show a strong direct correlation between YY1 and SOX2 expressions (R^2^ = 0.8). These associations were the initial basis for the distinctions among the four tiers. The tiers were then further subdivided based on the specific frequency of expression patterns of BMI1 and OCT4. When tiers three and four are grouped together, such that the results from liver, stomach, renal, pancreatic, urothelial cancers, lymphoma and melanoma are pooled, there is also a strong inverse correlation between YY1 and BMI1 expressions (R^2^ = −0.7). YY1 expression correlates strongly and differentially with the CSC markers’ expression in the different tiers.

## Conclusions, implications and speculations

YY1 frequency of expression was associated with the SOX2, BMI1 and OCT4 frequency of expression across many cancers, though the type of association varied. The differential patterns of the four markers’ expressions among the different tiers can be explained by the dual nature of YY1’s activities as both an activator and a repressor and in a direct or an indirect manner. We have examined the putative regulatory regions (promoters) of BMI1, SOX2, POU5F1 (OCT4) and YY1 for potential YY1 binding sites and reciprocal control of each other in a combinatorial matrix by using SABioscience’s Text-Mining Application [140] and data from the University of California, Santa Cruz (UCSC) Genome Browser [141] in order to define predicted binding sites associations of transcription factors to their regulatory regions. Our analysis demonstrated the presence of putative YY1 binding sites on all of the regulatory regions of the interrogated transcription factors (i.e., BMI1, SOX2, OCT4) including YY1 itself. However, none of the putative transcription binding sites for BMI1, SOX2, OCT4 were found on the YY1 or on each other’s regulatory regions. Noteworthy, a strong association (predicted binding site) was noted between NF-κB on the YY1 regulatory region and also between YY1 on the BMI1 promoter, suggesting a plausible transcriptional control by these factors.

The transcriptional association of YY1 on the regulatory regions of BMI1, SOX2, OCT4 and YY1 suggests a multi-dynamic regulatory control of expression. There is an NF-κB-mediated induction of expression of YY1 that can: a) inhibit the transcription of BMI1 and increase the expression of SOX2, resulting in up-regulation of OCT4 (e.g., Tier 1, Tier 2 and Tier 4 of the clustered groups); or b) activate the transcription of BMI1 and decrease the expression of SOX2, resulting in down-regulation of OCT4; or c) directly activate transcription of OCT4 (e.g., Tier 3 of clustered groups). Overall, the majority of the malignancies examined in this study have YY1 as a potential transcriptional repressor acting on CSCs-associated transcription factors.

It is important to note that the association of YY1 and CSC transcription factors was based on analysis of expression patterns on whole tumor tissues and not on the CSC subsets. Clearly, additional studies are necessary to determine whether the associations seen on whole tumor tissues is also found in the CSC subsets of the various cancers examined herein. The validation of YY1’s association with CSC markers and their functional roles in CSC regulation may provide new insights on the role of YY1 in carcinogenesis and its potential as a therapeutic target.

## Additional file

Additional file 1: Figure S1. Clustered Expression Association. Results were cluster-associated in 4 groups (tiers) based on the percentage of positive staining (Frequency of protein expression). Green boxes, ≧ 50 % positive staining. Blue boxes, <50 % positive staining. (JPG 66 kb)

## Abbreviations

Akt: protein kinase B
BMI1: B cell-specific Moloney murine leukemia virus insertion site 1
CSCs: cancer stem cells
EMT: epithelial-mesenchymal transition
NF-κB: nuclear factor kappa-B
PI3K: phosphatidylinositol 3-kinase
POUF51: POU class 5 homeobox 1
SOX2: sex determining region Y-box 2
YY1: Ying Yang 1
