# Supplementary figures and images for: Yin Yang 1 is associated with cancer stem cell transcription factors (SOX2, OCT4, BMI1) and clinical implication
Source: J Exp Clin Cancer Res. 2016 May 25;35:84. doi: 10.1186/s13046-016-0359-2 (PMC4881184; doi:10.1186/s13046-016-0359-2)

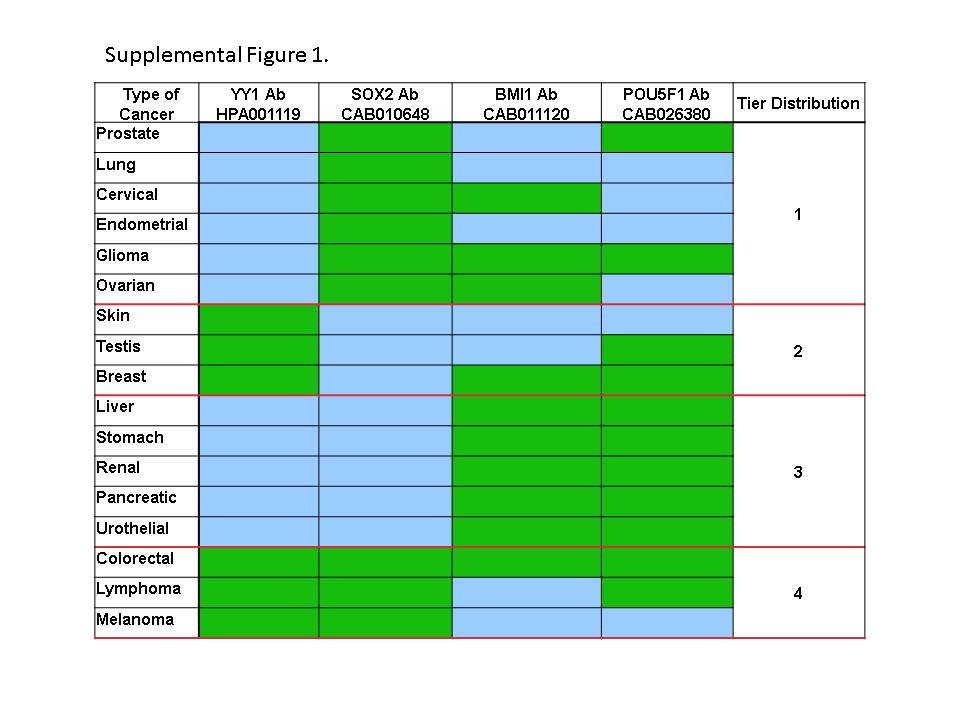

Supplement: Additional file 1: Figure S1. — Clustered Expression Association. Results were cluster-associated in 4 groups (tiers) based on the percentage of positive staining (Frequency of protein expression). Green boxes, ≧ 50 % positive staining. Blue boxes, <50 % positive staining. (JPG 66 kb) [file 13046_2016_359_MOESM1_ESM.jpg]
